# The Relation of CUN-BAE Index with Body Mass Index and Waist Circumference in Adults Aged 50 to 85 Years: The MCC-Spain Study

**DOI:** 10.3390/nu12040996

**Published:** 2020-04-03

**Authors:** Veronica Davila-Batista, Antonio J. Molina, Tania Fernández-Villa, Dora Romaguera, Beatriz Pérez-Gómez, Laura Vilorio-Marqués, Trinidad Dierssen-Sotos, Jone M. Altzibar, Victor Moreno, Eva Ardanaz, Inmaculada Salcedo-Bellido, Guillermo Fernández-Tardon, Rocio Capelo, Dolores Salas, Rafael Marcos-Gragera, José María Huerta, Silvia de Sanjosé, María Ángeles Sierra, José M. Canga-Presa, Ines Gómez-Acebo, Pilar Amiano, Marina Pollan, Nuria Aragones, Gemma Castaño-Vinyals, Manolis Kogevinas, Vicente Martín, on behalf of MCC-Spain study group

**Affiliations:** 1 Research Group on Gene-Environment Interactions and Health (GIIGAS), Instituto de Biomedicina (IBIOMED), University of León, 24071 León, Spain; 2 CIBER Epidemiología y Salud Pública (CIBERESP), 28029 Madrid, Spain; 3 Nutritional Methodology and Biostatistics Group, International Agency for Research on Cancer, 150 cours Albert Thomas, 69372 Lyon, France; 4 ISGlobal, 08003 Barcelona, Spain; 5 CIBER Fisiopatología de la Obesidad y Nutrición (CIBER-OBN), 28029 Madrid, Spain; 6 Instituto de Investigación Sanitaria de Palma (IdISPa) – Hospital Universitario Son Espases, 07120 Palma de Mallorca, Spain; 7 Environmental and Cancer Epidemiology Area, National Center of Epidemiology, Instituto de Salud Carlos III, 28029 Madrid, Spain; 8 Cancer Epidemiology Research Group, Oncology and Hematology Area, IIS Puerta de Hierro, 28222 Madrid, Spain; 9 Grupo de Investigación en Neoplasias Hematológicas, Instituto de Investigación Sanitaria Principado de Asturias (ISPA) and Fundación para la investigación Biosanitaria (FINBA), 33011 Oviedo, Spain; 10 Division of Epidemiology and Computational Biology, School of Medicine, University of Cantabria, 39011 Santander, Spain; 11 Public Health Division of Gipuzkoa, BioDonostia Research Institute, Osakidetza-Basque Health Service, Directorate General, 20014 San Sebastian, Spain; 12 Oncology Data Analytics Program, Catalan Institute of Oncology (ICO) and Oncobell Program, Bellvitge Biomedical Research Institute (IDIBELL), Hospitalet de Llobregat, 08908 Barcelona, Spain; 13 Department of Clinical Sciences, Faculty of Medicine, University of Barcelona, 08036 Barcelona, Spain; 14 IdiSNA, Navarra Institute for Health Research, 31008 Pamplona, Spain; 15 Preventive Medicine and Public Health Department, University of Granada and Instituto de Investigación Biosanitaria de Granada, Hospitales Universitarios de Granada, 18071 Granada, Spain; 16 Instituto de Investigación Sanitaria del Principado de Asturias (ISPA) and IUOPA, Universidad de Oviedo, 33006 Oviedo, Spain; 17 Centro de Investigación en Recursos Naturales, Salud, y Medio Ambiente (RENSMA), University of Huelva, 21071 Huelva, Spain; 18 Fundación para el Fomento de la Investigación Sanitaria y Biomédica de la Comunidad Valenciana (FISABIO), 46020 Valencia, Spain; 19 Epidemiology Unit and Girona Cancer Registry (UERCG), Oncology Coordination Plan (PDO), Department of Health, Autonomous Government of Catalonia, 17071 Girona, Spain; 20 Department of Epidemiology, Murcia Regional Health Council, IMIB-Arrixaca, 30007 Murcia, Spain; 21 Servicio de Cirugía General y Aparato Digestivo, Complejo Asistencial Universitario de León, 24001 León, Spain; 22 Epidemiology Section, Public Health Division, Department of Health of Madrid, 28035 Madrid, Spain; 23 IMIM (Hospital Del Mar Medical Research Institute), 08003 Barcelona, Spain; 24 Universitat Pompeu Fabra (UPF), 08003 Barcelona, Spain

**Keywords:** nutritional status, older adults, anthropometric measures, obesity, body site, agreement

## Abstract

*Backgound*: Traditional anthropometrics such as body mass index (BMI) or waist circumference (WC) do not fully capture the complex biology of body fat (BF) in the elderly. The Clinica Universidad de Navarra-Body Adiposity Estimator (CUN-BAE) index, based on BMI, is proposed as a better indicator of BF. However, its relation with BMI is not clear. The aim was to compare the agreement between CUN-BAE, BMI, and WC in those aged ≥50 years. *Methods*: A cross-sectional sample of 3153 Caucasian healthy adults was taken from the MCC-Spain study. The Pearson’s correlation and its 95% confidence interval (CI), adiposity distribution, and Kappa Index (95%CI) were calculated. *Results*: The correlation of CUN-BAE with WC is 0.18 (95%CI 0.14–0.21) and that with BMI is moderate (r 0.58; 95%CI 0.55–0.60), but both increased strongly by sex. Agreement (normal weight/overweight/obesity) of CUN-BAE with BMI is 7% and with WC is 18%. *Conclusions*: The correlation and the degree of agreement of CUN-BAE with BMI and WC are low in individuals aged over 50, but it is higher by sex. Thus, this different criterion of obesity may have clinical applications. More studies with a gold standard are needed to evaluate the CUN-BAE in elderly adults.

## 1. Introduction

The obesity and overweight epidemic affected more than 2100 million people in the world in 2016 [1]. It is estimated that this prevalence is increasing in all populations, and it particularly affects older adults [1]. In fact, excessive body fat (BF) is considered to be one of the metabolic disorders with the most serious consequences [2,3].

The most commonly used estimator of BF is body mass index (BMI), which reasonably predicts health risks in the global population. However, BMI is being judged due to its low predictive sensitivity, as half of people with a high BF% are not identified as obese by BMI [4]. Even the use of BMI has been discouraged for the elderly [5,6], because the biology of BF is influenced by age, sex, and race [7,8]. Therefore, this misrepresentation may have consequences when establishing the risk of disease related to excess BF [9], since nearly 40% of deaths and disability-adjusted life-years attributed to high body mass index (BMI) occurred in individuals who were not obese (BMI < 30 kg/m^2^) [2]. Another usual anthropometric measure is waist circumference (WC), as this estimator of abdominal obesity, closer to visceral fat, has been suggested for the assessment of cardiometabolic risk. However, it is not clear whether WC is more useful than BMI, so their independent use has not been recommended [10,11].

Therefore, different anthropometric alternatives to measure BF have been proposed [12]. Among these, an index that deserves special consideration is the Clinica Universidad de Navarra—Body Adiposity Estimator (CUN-BAE). This is an adiposity estimator based on BMI, sex, and age in Caucasian adults [13,14]. The CUN-BAE index was proposed as a better indicator of BF than BMI or WC in adults to predict cardiovascular disease and other outcomes, as well as, it might be better able to identify metabolically unhealthy individuals [15,16,17,18,19]. Therefore, the Spanish Society for the Study of Obesity (SEEDO) has promoted the use of CUN-BAE in strategies of obesity prevention since 2016 [11]; however, the evidence on the CUN-BAE is not convincing.

CUN-BAE shows a strong correlation with BF% measured by direct techniques in all adults, especially in women, but studies in older adults are limited [15,20,21]. Also, the relation and concordance between the CUN-BAE and traditional anthropometrics are not clear, especially the relation with BMI as a component within the equation.

The aim of this study is to compare the relations between CUN-BAE, BMI, and WC in individuals aged over 50 years.

## 2. Materials and Methods

### 2.1. Study Design

The study population was selected from among the controls recruited in the multicentre case-control MCC-Spain project. The MCC-Spain was a population-based study carried out in 12 Spanish provinces from eight autonomous communities (Asturias, Barcelona, Cantabria, Girona, Granada, Guipúzcoa, Huelva, León, Madrid, Murcia, Navarra, and Valencia) between 2008 and 2013. The aim of the MCC-Spain study was to evaluate the role of environmental exposures and genetic factors in relevant tumours in the Spanish population [22].

The MCC-Spain healthy control group (4098 controls recruited) was selected from the general population and frequency matched to the cases according to age, sex, and region. The members of the control group were randomly selected from people enrolled in primary care centres within the reference areas recruited and were invited to participate in the study. They included individuals aged between 24 and 85 years who were able to answer an epidemiological questionnaire and had resided for at least six months in the catchment areas of collaborating hospitals. More details of the project can be found at http://www.mccspain.org and in a previous article published by the MCC-Spain project [22].

For the present study we included an MCC-Spain control subsample of 3153 Caucasian subjects with valid anthropometric information, with BMI greater than or equal to 18.5 kg/m^2^, and aged between 50 and 85 years (78% of the 4098 control subjects, supplementary Figure S1).

### 2.2. Data Collection

The epidemiological information was collected using a structured, computerised questionnaire by trained personnel following the study protocol. A questionnaire on the socio-demographic, lifestyle, and medical history of personal antecedents, occupational and residential history, dietary intake, and anthropometric measurements, which included self-reported height and weight, was collected [22].

#### Anthropometric Assessment

BMI was calculated as weight in kilograms divided by the square of height in metres. Individuals were classified as normal weight (BMI of 18.5–24.9 kg/m^2^), overweight (25–29.9kg/m^2^) or obese (≥30 kg/m^2^) [23]. WC was measured twice by the interviewers with the subject standing using a flexible tape measure in centimetres according to a standardised protocol, including a third measurement if the difference between them was over 0.5 cm. An at-risk waistline was 80–88 cm in women and 94–102 cm in men; abdominal obesity was >102 cm in men and >88 cm in women [23].

The CUN-BAE was calculated using the equation, proposed by Gómez-Ambrosi et al. [15]:

%BF = −44.988 + (0.503 × age) + (10.689 × sex) + (3.172 × BMI) − (0.026 × BMI^2^) + (0.181 × BMI × sex) − (0.02 × BMI × age) − (0.005 × BMI^2^ × sex) + (0.00021 × BMI^2^ × age)

where age is measured in years and sex is codified as men = 0 and women = 1.

For the CUN-BAE, the category percentage of normal body fatness is ≤20% in men and ≤30% in women; overweight is 20–25% in men and 30–35% in women; and obesity is >25% BF in men and >35% in women [4,15,24].

### 2.3. Statistical Analysis 

A descriptive analysis of the characteristics of the participants was carried out using the arithmetic mean and the standard deviation (SD) for numerical variables, and the absolute and relative frequencies (%) of the categorical variables. 

As quantitative variables, we evaluated the correlations between the three studied measures—BMI, WC, and CUN-BAE—using Pearson’s correlation coefficient (r) and the 95%CI. 

As categorical variables for the criteria used for each index, we analysed the distribution of BMI, WC, and CUN-BAE through descriptive statistics and by comparing the distributions of individuals according to the different criteria of adiposity (normal weight/overweight/obesity). To assess the degree of agreement classifying individuals as normal weight, overweight, and obese between the two forms of rating, we calculated a weighted Kappa Index coefficient (95% CI), with majority agreement weights 0/0.5/1. We also calculated a lineal Kappa Index (95% CI) as non-obesity vs. obesity. All analyses were stratified by sex. Data analysis was carried out using the Stata/SE 15 software package (Stata Corp./SE, College Station, TX, USA).

### 2.4. Ethical Obligations

The protocol of the study was approved by the ethics committees at all participating institutions. The study was carried out in accordance with the ethical standards of the Declaration of Helsinki and the legal regulations on data privacy. The database was registered in the Spanish Agency for Data Protection, number 2102672171.

## 3. Results

A total of 3153 healthy individuals were included; 1836 were men and 1317 were women. The mean ages of these two groups were 67.6 years (SD 7.9) and 64.7 years (SD 9.2), respectively. Characteristics of the study population are shown in Table 1. 

Among the anthropometric measures included, the BMI mean was 27.4 kg/m^2^ (SD 3.8) in men and 26.5 kg/m2 (SD 4.8) in women. For WC, the mean was 102.0 cm (SD 10.7) in men and 91.4 cm (SD 13.3) in women. For CUN-BAE, the mean was 29.9 BF% (SD 4.2) in men and 40.2 BF% (SD 5.3) in women.

**Figure 1:**
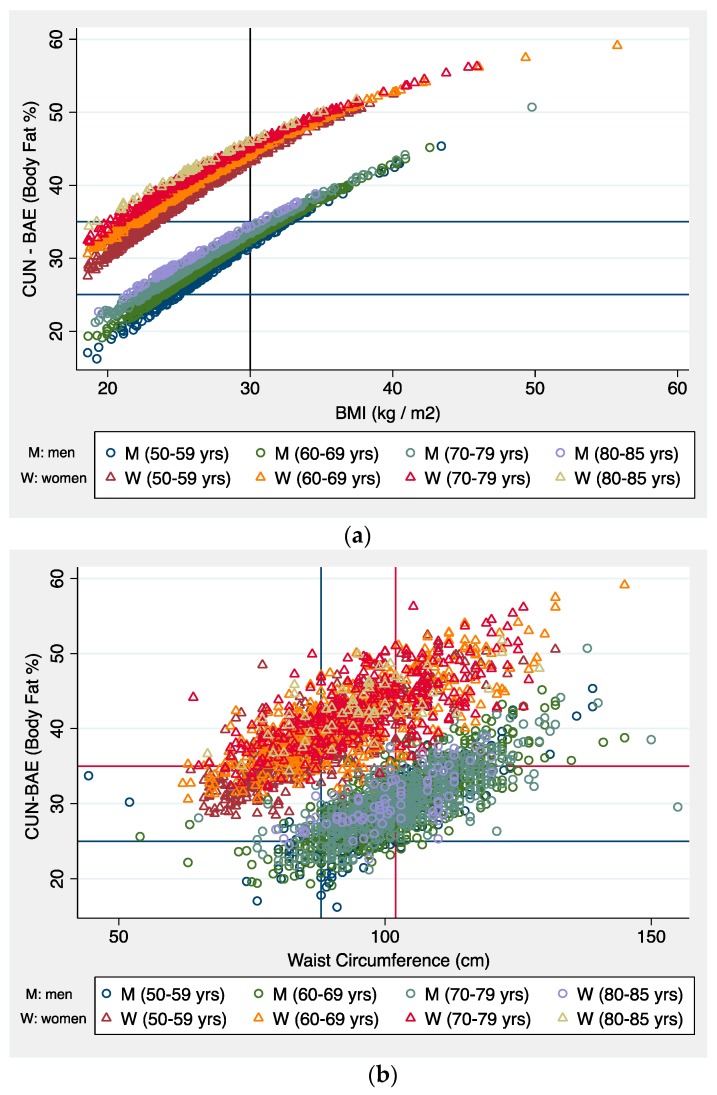
Distributions of CUN-BAE against traditional anthropometric measures. (**a**) Distribution of body mass index and CUN-BAE; (**b**) Distribution of waist circumference and CUN-BAE. Note: yrs, years.

Figure 1 shows the distributions of CUN-BAE with a) BMI and b) waist circumference, observed for two separate groups according to sex. We observed a greater dispersion between WC and CUN-BAE than between BMI and CUN-BAE. 

Table 2 shows the correlation coefficients among the different indices. The overall correlation between BMI and CUN-BAE was medium (r = 0.58); when sex was taken into account, it increased to above r ≈ 0.96 for both sexes. The overall correlation between WC and CUN-BAE was very low (r = 0.18), and we observed that it also improved when taking sex into account (r ≈ 0.76). 

When the analysis was restricted according to BMI, a comparison of CUN-BAE and BMI showed that among the obese, the correlation (r = 0.56) was slightly higher than that in individuals with a BMI of ≤30 kg/m^2^ (r = 0.31), and when taking sex into account, the correlation was strong (r = 0.93) and the difference was tempered. The results were similar when stratification was performed based on abdominal obesity. The correlations between CUN-BAE, body mass index, and waist circumference by age groups are show in supplementary materials Table S1. 

Table 3 shows the distribution of the study population according to adiposity criteria for the anthropometric measures. We observed large differences in the distribution of the categories (normal weight/overweight/obesity) according to the anthropometric measure used. The prevalences of obesity observed with each of the criteria used were 21.1%, 49.7%, and 86.0% for BMI, WC, and CUN-BAE, respectively, in the overall analysis. In all cases, the relative frequency of subjects with a high BF was higher with CUN-BAE than with BMI and WC. 

Table 4 shows the degree of agreement between the different measures. The agreement between BMI and CUN-BAE for the classification of normal weight, overweight, and obesity was very low (kappa index 0.068: 95%CI 0.065–0.074) and showed no improvement when analysed by sex. Between WC and CUN-BAE, the weighted agreement was 0.180 (CI95% 0.171–0.199); interestingly, the kappa index was slightly better in women than in men, but in none of the cases did it reach an acceptable value (<0.25).

## 4. Discussion

The correlations between the two most commonly used anthropometric measures (BMI and WC) with the CUN-BAE body fat estimator were not good in individuals over 50 years, and neither was suitable in terms of the degree of agreement for overweight/obesity. To date, the relation between CUN-BAE and BMI in older adults is unclear, and misclassification of obesity could have practical implications. 

That BMI and direct measurements of BF% are not strongly correlated has been known for over two decades [25,26]. In the general population, CUN-BAE has shown stronger correlations with direct measures of BF% than other anthropometric measures such as BMI, WC, body adiposity index (BAI), or body shape index (ABSI) [12,17,19,20,21]. CUN-BAE—based on BMI, sex, and age—has been shown to be an independent measure of BMI [16]. 

In the current study, the correlation between CUN-BAE and BMI was very low. It was weaker than those observed in other studies in the general population [15,16,17,19,20,27]. The results of this study are original, as well as, CUN-BAE is an indirect index of BF and we do not have a gold standard, which means that a low correlation does not imply that BMI and WC are weaker indicators.

However, to interpret the relation between CUN-BAE and BMI we should remember that CUN-BAE is an index, based on BMI, which includes a quadratic function to consider nonlinear relationships. Then, the association between BF% and BMI is determined by the effect of sex, age, and ethnicity [7,8], and the different low correlations observed may be due to participants’ age ranges being heterogeneous in the different studies, with young adults included in the majority of other authors’ works. Further, only one small study (sample size of 40) evaluated the CUN-BAE in older persons [27]. It is known that older individuals have a higher proportion of fat mass and that as a result, the correlation between WC and/or BMI and percentage BF decreases with age [28,29]. In fact, BMI is not a recommended anthropometric measure for evaluating adiposity in elderly individuals [5]. Additionally, the changes in body composition as redistribution of fat from the subcutaneous tissue with age, makes it very difficult to determine an optimal anthropometric index in elderly.

Additionally, the correlation improved greatly when the sample was stratified by sex; this coincides with the results of other studies [16,19,20,27]. Thus, by sex, the BMI and CUN-BAE correlation is strong (r > 0.94), while between WC and CUN-BAE, the correlation is somewhat lower (r 0.76–0.80). This is linked to the different distribution and biology of adiposity according to the sex of the individuals [30]. In assessing the relation between WC and CUN-BAE stratified by a BMI of 30 kg/m^2^, we were struck by the negative correlation in the global values. This may be due to ecological bias due to adding two clearly differentiated groups such as men and women, as shown in Figure 1 of the results. This further supports the need to differentiate by sex when we talk about BF. 

The most useful aspect of anthropometric indices is the diagnosis of overweight and obesity. In this line, CUN-BAE categorises a greater number of subjects with obesity than do BMI or WC (86.01 vs. 21.06 vs. 49.73). Following these criteria, there is great disagreement in the classification (weighted kappa index values of CUN-BAE vs. BMI and WC of 0.068 and 0.174). In this way, other authors have reported that the obesity prevalences determined by BMI and %BF showed great differences [4,18,29]. 

There is convincing evidence for BMI obesity standards and their strong association with long-term outcome and comorbidity. With regard to the cut-off points for CUN-BAE, the most widely used criteria in the scientific literature are >20% as overweight and >25% BF as obese in men and >30% as overweight and >35% BF as obese in women [4,15,24]. However, there is no consensus for categorising adiposity based on BF percentage [31]. A cut-off point that leaves no one in the normal is not useful to assess cardiovascular risk and mortality. More studies are necessary to establish the ideal cut-off point for body fat associated with optimum health [23,31,32].

This could have great repercussions in the Public Health, since it a large increase in the number of people diagnosed with obesity and maybe overloading health systems. On the other side, many of these subjects will be obese with normal weight with metabolically unhealthy [33]; and the use of CUN-BAE can contribute to improve more healthy lifestyle of the individual. Regardless of the diagnostic tool used, CUN-BAE, BMI or WC, healthy lifestyle with proper diet and physical exercise are general recommendations for all population.

This study, like all observational studies, has strengths and limitations. As the main strengths, we included a large sample of healthy subjects with large geographical variability across Spain. Moreover, we only included Caucasian subjects, since the ethnic group is relevant to calculating the BF% and other studies did not consider it. 

A limitation of this study was the bias that accompanies any cross-sectional observational study. Also, we did not have a direct measurement method of BF to deploy as a gold standard; thus, we can only evaluate the relative usefulness of the different measures. Another possible limitation is that the CUN-BAE equation was validated using a sample with other purposes, which had a higher proportion of sedentary participants. Nevertheless, the CUN-BAE presented high correlation and metrics with BF measured by dual energy X-ray absorptiometry (DXA) in other studies [12,34]. While, the WC measurements were collected by qualified individuals who were trained for this purpose, height and weight were self-reported. 

Thus, it is necessary to know the CUN-BAE better before recommending this adiposity estimator in regular physical examinations, especially in the elderly. More studies that will help to validate the CUN-BAE in a representative sample of the Spanish Caucasian population are required, and they should also assess its usefulness in determining the risk of developing diseases associated with adiposity.

## 5. Conclusions

The correlation of the percentage of BF measured by CUN-BAE with that according to BMI and WC is very low in individuals aged over 50. However, this correlation is higher by sex. Similarly, the degrees of agreement in assessing normal weight, overweight, and obesity among the three indices studied are low. Further studies are needed to determine the usefulness of the available indicators in estimating the percentage of body fat in older subjects.

## Figures and Tables

**Table 1:** Characteristics of the study population.

Variables	Men (*n* = 1836)		Women (*n* = 1317)	
	*n*	%	*n*	%
Age				
50–59 years	279	15.2	432	32.8
60–69 years	787	42.9	446	33.9
70–79 years	654	35.6	357	27.1
80–85 years	116	6.3	82	6.2
(mean, SD)	67.6 (7.9)		64.7 (9.2)	
Educational Level				
Less than primary	342	18.6	289	21.9
Primary school	624	34.0	460	34.9
Secondary education	489	26.6	363	27.6
University	381	20.8	205	15.6
Marital status				
Single	87	4.7	112	8.5
Married or partner	1582	86.3	857	65.1
Separated or divorced	78	4.3	87	6.6
Widowed	87	4.7	260	19.8
Smoking Status				
Never	505	27.6	860	65.3
Former	933	51.0	249	18.9
Smoker	390	21.3	208	15.8
Hypertension (yes)	876	47.8	490	37.3
Diabetes (yes)	322	18.6	129	10.2
Height (cm) (mean, SD)	169.6 (6.5)		158.5 (6.4)	
Body Mass Index				
<25 kg/m^2^	483	26.3	570	43.3
25–30 kg/m^2^	954	52.0	482	36.6
≥30 kg/m^2^	399	21.7	265	20.1
(mean, SD)	27.4 (3.7)		26.5 (4.7)	
CUN-BAE				
M ≤ 20 or W ≤ 30 BF%	12	0.7	18	1.4
M 20–25 or W 30–35 BF%	199	10.8	212	16.1
M > 25 or W > 35 BF%	1625	88.5	1087	82.5
(mean, SD)	29.9 (4.2)		40.2 (5.3)	
Waist circumference				
M < 94 or W < 80 cm	426	23.2	287	21.8
M 94–102 or W 80–88 cm	578	31.5	294	22.3
M > 102 or W > 88 cm	832	45.3	736	55.9
(mean, SD)	102.0 (10.7)		91.4 (13.3)	

BF, body fat; CUN-BAE, Clínica Universidad de Navarra—Body Adiposity Estimator; M, men; W, women.

**Table 2:** Correlations between CUN-BAE, body mass index, and waist circumference.

Type of Measure	All			Men			Women		
	*n*	Pearson’s r	(95%CI)	*n*	Pearson’s r	(95%CI)	*n*	Pearson’s r	(95%CI)
**BMI vs. CUN-BAE**	3153	0.576	0.552; 0.599	1836	0.972	0.970; 0.975	1317	0.962	0.958; 0.966
BMI < 30 kg/m^2^	2489	0.313	0.277; 0.348	1437	0.936	0.930; 0.942	1052	0.937	0.929; 0.944
BMI ≥ 30 kg/m^2^	664	0.557	0.502; 0.607	399	0.974	0.968; 0.979	265	0.960	0.950; 0.969
WC no abdominal obesity	1585	0.260	0.213; 0.305	1004	0.937	0.929; 0.944	581	0.925	0.912; 0.936
WC abdominal obesity	1568	0.515	0.477; 0.550	832	0.979	0.976; 0.982	736	0.964	0.959; 0.969
**WC vs. CUN-BAE**	3153	0.180	0.146; 0.213	1836	0.756	0.736; 0.775	1317	0.768	0.745; 0.790
BMI < 30 kg/m^2^	2489	−0.101	−0.140; −0.062	1437	0.629	0.597; 0.660	1052	0.643	0.606; 0.677
BMI ≥ 30 kg/m^2^	664	−0.059	−0.134; −0.017	399	0.574	0.504; 0.636	265	0.533	0.441; 0.614
WC no abdominal obesity	1585	−0.395	−0.435; −0.352	1004	0.490	0.442; 0.536	581	0.534	0.473; 0.590
WC abdominal obesity	1568	−0.076	−0.125; −0.026	832	0.636	0.594; 0.675	736	0.616	0.569; 0.659
**BMI vs. WC**	3153	0.723	0.706; 0.739	1836	0.742	0.720; 0.761	1317	0.752	0.728; 0.775

BMI, body mass index; CUN-BAE, Clínica Universidad de Navarra—Body Adiposity Estimator; WC (waist circumference): abdominal obesity determined by WC > 102 cm in men and WC > 88 cm in women.

**Table 3:** Distribution of individuals according to the different obesity criteria.

Type of Measurement		CUN-BAE			
		Normal Fat	Overweight	Obesity	Total
All	Body Mass Index				
	normal weight	30	410	613	1053 (33.4%)
	overweight	0	1	1435	1436 (45.6%)
	obesity	0	0	664	664 (21.1%)
	Waist circumference				
	normal waist	27	295	391	713 (22.6%)
	at-risk waistline	1	87	784	872 (27.7%)
	abdominal obesity	2	29	1537	1568 (49.7%)
	Total	30 (1.0%)	411 (13.0)	2712 (86.0)	3153
Men	Body Mass Index				
	normal weight	12	198	273	483 (26.3%)
	overweight	0	1	953	954 (52.0%)
	obesity	0	0	399	399 (21.7%)
	Waist circumference				
	normal waist	12	153	261	426 (23.2%)
	at-risk waistline	0	41	537	578 (31.5%)
	abdominal obesity	0	5	827	832 (45.3%)
	Total	12 (0.7)	199 (10.8)	1625 (88.5)	1836
Women	Body Mass Index				
	normal weight	18	212	340	570 (43.3%)
	overweight	0	0	482	482 (36.6%)
	obesity	0	0	265	265 (20.1%)
	Waist circumference				
	normal waist	15	142	130	287 (21.8%)
	at-risk waistline	1	46	247	294 (22.3%)
	abdominal obesity	2	24	710	736 (55.9%)
	Total	18 (1.3%)	212 (16.1%)	1087 (82.5%)	1317

CUN-BAE, Clínica Universidad de Navarra—Body Adiposity Estimator; normal fat in men, ≤20 body fat percentage, women, ≤30%; overweight men, 20–25%, women 30–35%; and obesity in men, >25%, women, >35%. Body mass index: normal weight, <25 kg/m^2^; overweight, 25–30 kg/m^2^; and obesity, ≥30 kg/m^2^. Waist circumference: normal waist in men, <94 cm, women, <80 cm; at-risk waistline in men, 94–102 cm, women, 80–88 cm; and abdominal obesity in men, >102 cm, women, >88 cm.

**Table 4:** Degrees of agreement of the categories of adiposity among anthropometrics measures.

Type of Measurement		CUN-BAE	
		Weighted Kappa Index (CI 95%) (Normal Weight, Overweight vs. Obese)	Lineal Kappa Index (CI 95%) (Non-Obesity vs. Obesity)
BMI	All	0.068 (0.065–0.074)	0.083 (0.074–0.093)
	Men	0.061 (0.058–0.063)	0.070 (0.059–0.081)
	Women	0.076 (0.069–0.085)	0.101 (0.085–0.118)
WC	All	0.180 (0.171–0.199)	0.238 (0.215–0.261)
	Men	0.141 (0.139–0.151)	0.184 (0.159–0.209)
	Women	0.251 (0.242–0.270)	0.337 (0.294–0.381)

CUN-BAE, Clínica Universidad de Navarra—Body Adiposity Estimator; normal fat in men, ≤20 body fat percentage, women, ≤30%; overweight in men, 20–25%, women, 30–35%; and obesity in men, >25%, women, >35%. BMI (Body Mass Index): normal weight, <25 kg/m^2^; overweight, 25–30 kg/m^2^; and obese, ≥30 kg/m^2^. WC (Waist circumference): normal waist in men, <94 cm, women, <80 cm; at-risk waistline in men, 94–102 cm, women, 80–88 cm; and abdominal obesity in men, >102 cm, women, >88 cm.

## Supplementary Materials

The following are available online at https://www.mdpi.com/2072-6643/12/4/996/s1, Figure S1: Flowchart of participant inclusion in the study, Table S1: Correlations between CUN-BAE, body mass index, and waist circumference by age group.
