# Supplementary material for: The Relation of CUN-BAE Index with Body Mass Index and Waist Circumference in Adults Aged 50 to 85 Years: The MCC-Spain Study
Source: Nutrients. 2020 Apr 3;12(4):996. doi: 10.3390/nu12040996 (PMC7231053; doi:10.3390/nu12040996)
Supplement: Supplementary file 1 [file nutrients-12-00996-s001.pdf]

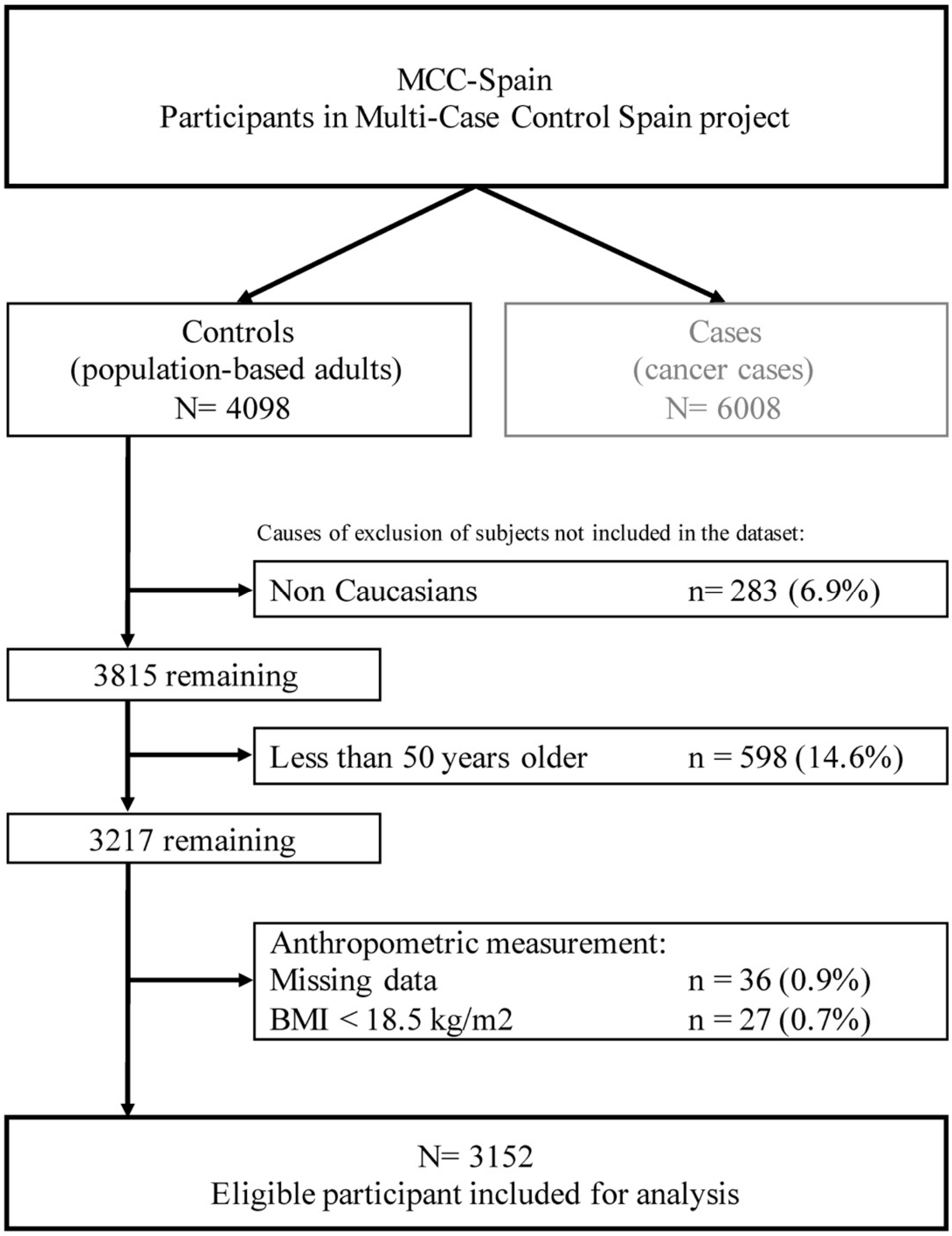

Figure S1. Flowchart of participant inclusion in the study.

Table S1. Correlations between CUN-BAE, body mass index, and waist circumference by age group.

Type of measure	All			Men			Women		
	n	**Pearson's r**	**(95%CI)**	***n***	**Pearson's r**	**(95%CI)**	***n***	**Pearson's r**	**(95%CI)**
BMI vs CUN-BAE									
50–59 years	711	0.5539	0.501; 0.603	279	0.994	0.993; 0.996	432	0.992	0.991; 0.994
60–69 years	1233	0.5875	0.550; 0.623	787	0.996	0.995; 0.996	446	0.985	0.982; 0.988
70–79 years	1011	0.6065	0.566; 0.644	654	0.994	0.993; 0.995	357	0.990	0.988; 0.992
80–85 years	198	0.5545	0.450; 0.644	116	0.997	0.996; 0.998	82	0.996	0.994; 0.998
WC vs CUN-BAE									
50–59 years	711	0.1839	0.112; 0.254	279	0.709	0.645; 0.763	432	0.774	0.734; 0.810
60–69 years	1233	0.2176	0.164; 0.270	787	0.794	0.766; 0.818	446	0.779	0.740; 0.813
70–79 years	1011	0.1602	0.100; 0.220	654	0.738	0.701; 0.771	357	0.703	0.646; 0.752
80–85 years	198	0.0659	-0.074 ; 0.203	116	0.704	0.599; 0.786	82	0.673	0.533; 0.776
BMI vs WC									
50–59 years	711	0.7603	0.727; 0.790	279	0.715	0.653; 0.768	432	0.773	0.732; 0.808
60–69 years	1233	0.7630	0.739 ; 0.785	787	0.791	0.763; 0.816	446	0.781	0.742; 0.814
70–79 years	1011	0.6712	0.636; 0.704	654	0.737	0.700; 0.770	357	0.690	0.631; 0.740
80–85 years	198	0.6342	0.543; 0.711	116	0.702	0.596; 0.784	82	0.661	0.519; 0.768

BMI, body mass index; CUN-BAE, Clínica Universidad de Navarra—Body Adiposity Estimator; WC, waist circumference.
